# A new replacement name for *Tropidocephala speciosa* Ding, 2006 (Hemiptera, Fulgoromorpha, Delphacidae)

**DOI:** 10.3897/zookeys.462.8332

**Published:** 2014-12-10

**Authors:** Hai-Yan Sun, Lin Yang, Xiang-Sheng Chen

**Affiliations:** 1 Institute of Entomology, Guizhou University, Guiyang, Guizhou, P.R. China, 550025; 2 Special Key Laboratory for Development and Utilization of Insect Resources, Guizhou University, Guiyang, Guizhou, P.R. China, 550025; 3 College of Animal Sciences, Guizhou University, Guiyang, Guizhou, P.R. China, 550025

**Keywords:** Hemiptera, Fulgoroidea, *Tropidocephala*, homonym, replacement name

## Abstract

A new replacement name is proposed for the species *Tropidocephala speciosa* Ding, 2006 (Hemiptera: Fulgoromorpha: Delphacidae: Tropidocephalini), preoccupied by *Tropidocephala speciosa* (Bierman, 1908): *Tropidocephala dingi* Sun, Yang & Chen, **nom. n.** = *Tropidocephala speciosa* Ding, 2006. The photographs and illustrations of this species are also provided.

## Introduction

The plant-hopper species *Tropidocephala speciosa* Ding, 2006 (Hemiptera: Fulgoromorpha: Delphacidae: Tropidocephalini) is found to be preoccupied by *Tropidocephala speciosa* (Bierman, 1908). The purpose of the present paper is to propose a replacement name, based on new material.

## Materials and methods

Morphological techniques and terminology follows Ding (2006). Dry specimens were used for the description and illustration. External morphology was observed under a stereoscopic microscope and characters were measured with an ocular micrometer. The genital segments of the examined specimens were macerated in 10% KOH and drawn from preparations in glycerin jelly using a Leica MZ 12.5 stereomicroscope. Photographs of the specimens were taken with a KEYENCE VHX-1000C. Illustrations were scanned with Canon CanoScan LiDE 200 and imported into Adobe Photoshop CS3 for labeling and fig composition.

Specimens examined are deposited in the Institute of Entomology, Guizhou University, Guiyang, Guizhou Province, China (IEGU).

## Nomenclatural changes and notes

### Tropidocephala dingi

Taxon classificationAnimaliaHemipteraDelphacidae

Sun, Yang & Chen nom. n.

Figs 1–12

Tropidocephala dingi , nomen novum for *Tropidocephala speciosa* Ding, 2006: 167, preoccupied by *Tropidocephala speciosa* (Bierman, 1908).

#### Remarks on nomenclatural change.

Melichar (1914) transferred *Orchesma speciosa* Bierman, 1908 to *Tropidocephala* as a new combination. Ding (2006) described a new species, *Tropidocephala speciosa*, and failed to recognize the homonym. Thus, the species *Tropidocephala speciosa* Ding, 2006 is a secondary homonym of the species *Tropidocephala speciosa* (Bierman, 1908). According to Article 60 and 57.3.2 of the ICZN, we propose a new replacement name *Tropidocephala dingi* nom. n. for *Tropidocephala speciosa* Ding, 2006.

#### Etymology.

This new name is based on the surname of the author of the junior homonym.

#### Distribution.

China (Hainan and Yunnan Province).

#### Specimens examined.

1♂1♀, Diaoluoshan National Natural Reserve, Hainan, 9-12 Apr. 2009, X.-H. Hou; 1♂1♀, Bawangling National Natural Reserve, Hainan, 24-28 Apr. 2009, X.-H. Hou; 2♂♂, Bawangling National Natural Reserve, Hainan, 6-7 Jan. 2011, J.-K. Long; 4♂♂2♀♀, Yinggeling National Natural Reserve, Hainan, 17-18 Apr. 2014, H.-Y. Sun; 1♂, Diaoluoshan National Natural Reserve, Hainan, 27 Apr. 2009, H.-Y. Sun.

**Figures 1–12.**
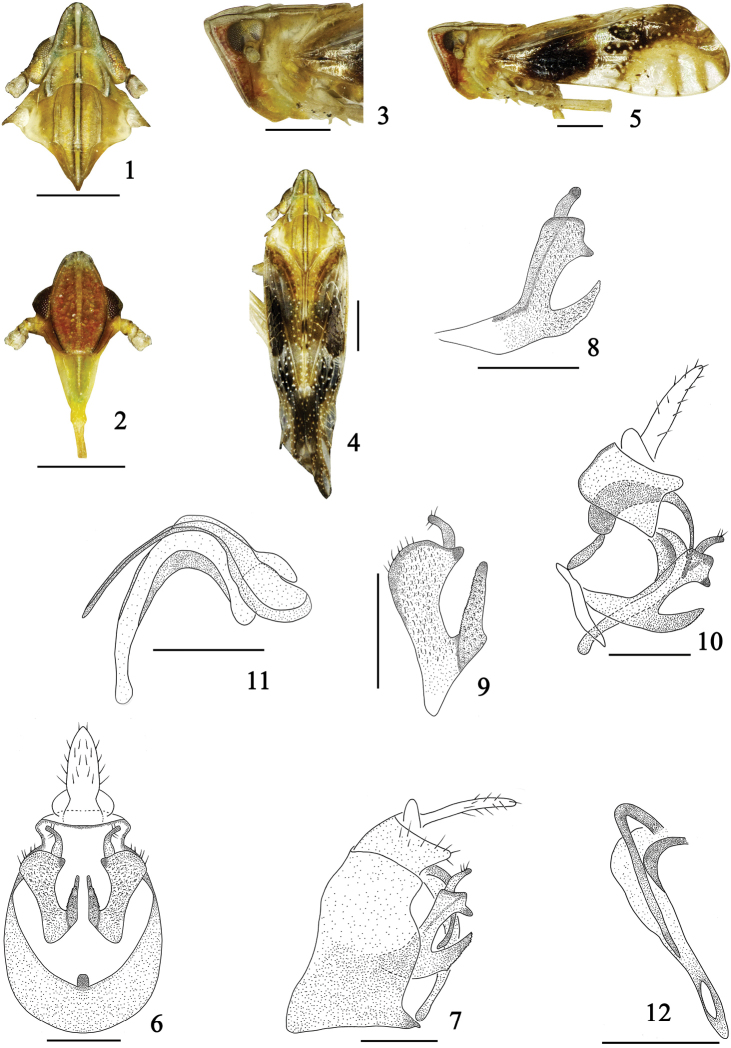
*Tropidocephala dingi* Sun, Yang & Chen, nom. n. **1** head and thorax, dorsal view **2** frons and clypeus **3** head and thorax, lateral view **4** male habitus, dorsal view **5** the same, lateral view **6** male genitalia, posterior view **7** the same, lateral view **8** genital style, lateral view **9** the same, posterior view **10** anal segment, aedeagus and genital style, left lateral view **11** aedeagus, lateral view **12** aedeagus, posterior view. Scale 0.5 mm (Figures **1–5**); 0.2 mm (Figures **6–12**).

## Supplementary Material

XML Treatment for Tropidocephala dingi
